# Seed Protein of Lentils: Current Status, Progress, and Food Applications

**DOI:** 10.3390/foods8090391

**Published:** 2019-09-04

**Authors:** Hamid Khazaei, Maya Subedi, Mike Nickerson, Cristina Martínez-Villaluenga, Juana Frias, Albert Vandenberg

**Affiliations:** 1 Department of Plant Sciences, University of Saskatchewan, Saskatoon, SK S7N 5A8, Canada (M.S.) (A.V.); 2 Food and Bioproduct Sciences, University of Saskatchewan, Saskatoon, SK S7N 5A8, Canada; 3 Institute of Food Science, Technology and Nutrition (ICTAN), Spanish National Research Council (CSIC), Jose Antonio Novais 10, 28040 Madrid, Spain (C.M.-V.) (J.F.)

**Keywords:** *Lens*, protein, amino acid, legume, functionality, bioactive peptides

## Abstract

Grain legumes are widely recognized as staple sources of dietary protein worldwide. Lentil seeds are an excellent source of plant-based proteins and represent a viable alternative to animal and soybean proteins for food processing formulations. Lentil proteins provide not only dietary amino acids but are also a source of bioactive peptides that provide health benefits. This review focuses on the current knowledge of seed protein, extraction and isolation methods, bioactive peptides, and food applications of lentil protein. Lentil is the most rapidly expanding crop for direct human consumption, and has potential for greater impact as a protein source for food processing applications. Improvements in lentil protein quality, amino acid composition, and processing fractions will enhance the nutritional quality of this rapidly expanding crop globally.

## 1. Introduction

The importance of food legumes in sustainable agriculture and food security is increasing worldwide. Cultivation of food legumes delivers generous environmental and economic benefits based on their ability to fix nitrogen to replace synthetic fertilizers and thereby reduce greenhouse gas emissions. In contrast with animal-based protein, which has a huge environmental cost, seeds of grain legumes are widely consumed as staple sources of dietary proteins. They are the main source of dietary protein for over one billion people and provide opportunities for greater use in new plant-based protein foods and animal feeds. Protein confers food value, handling properties, and gustatory qualities [1].

Cultivated lentil (*Lens culinaris* Medik.), one of the ancient crops, is a quick-cooking and nutritious staple legume grown in more than 70 countries and consumed globally in whole, dehulled, and split form [2]. Its seeds are lens-shaped and have a wide range of seed coat colours (green, tan, brown, gray, white, and black) and patterns (marbled, dotted, spotted, complex, and unpatterned [3]). The cotyledons can be yellow, red, or green. Red cotyledon lentils are a source of staple protein and nutritious food in many parts of the Indian subcontinent and eastern Mediterranean regions, where they are consumed mainly in dehulled form as split cotyledons [4]. The major commercial market classes of lentil are red (based on cotyledon colour of dehulled seeds) and green (based on seed coat colour). Large green lentils (with yellow cotyledon) are primarily marketed in Europe, and parts of the Middle East and South America, mostly consumed as whole seeds [5].

Lentil seeds are typically rich in protein, dietary fiber, complex carbohydrates, and essential micronutrients such as iron, zinc, and vitamin B complex [6]. Its seeds also have high antioxidant activity compared to other grain legume species mainly due to specific phenolic compounds [7]. Tahir et al. [8] reported only small differences between green and red lentils for protein concentration.

Lentil is in high demand globally and has, by far, the highest growth rate in consumption in comparison to other major pulses (Table 1). Since the beginning of the green revolution (1960–1965), the annual increase of production of faba bean and pea have not kept pace with the growth of the human population (2.4% annual average growth). Chickpea and common bean have increased at the same rate as population growth, while lentil production has grown at more than 10% per year, second only to soybean. The main reason for the high growth rate is presumed to be the fast cooking time of lentils relative to the other main pulses, in spite of the fact that lentil is often more expensive than most of the other pulses.

Nearly one third of the world’s population, particularly children in low-income countries, is protein deficient [10,11]. The high protein content of lentil, its fast-cooking time, and increased production are contributing to lentil gaining in importance as a staple food for combating human protein malnutrition globally. There is a great need to accelerate genetic improvement for high-yielding lentil cultivars with high quantity and quality of protein. This review focuses on the current knowledge of lentil protein quantity and quality, and the gap between current knowledge, and what knowledge is needed to alleviate nutritional and environmental concerns.

## 2. Protein Content

Like seeds of most cultivated legumes, lentils are a rich source of high-quality protein. Lentil seeds contain on average about 26% crude protein (see Table 2). Several studies have reported genetic variation for protein content in lentil seeds (Table 2). A Russian bulletin published in 1930 reported that the protein content among lentil varieties varied from 27.5%–31.7% [12]. In the 1970s, an evaluation of an extensive global collection of 1688 accessions of lentil for protein content, reported a greater range of protein content from of 23.4%–36.4% [13]. A subsequent evaluation reported an even wider range when a larger germplasm set (1816 accessions) was investigated [14]. Kumar et al. [15] reported a lower average protein content for lentil with broader variability among lentil species.

## 3. Lentil Seed Storage Proteins

Seed storage proteins of lentil are located in the cotyledons, representing up to 80% of total proteins. Seed storage proteins primarily provide nitrogen, carbon, and sulphur during seed germination and seedling growth and development [24]. They are also involved in plant defence mechanisms, e.g., for bruchids in legumes [25], and in antimicrobial activity (reviewed in Cândido et al. [26]). Seed storage proteins are classifiable based on their solubility in different solvents. The first report of protein components in lentil was that of Osborne and Campbell [27] who isolated globulins from lentil seeds. Later, Danielson [28] grouped the globulins into two classes, 7S (vicilin and convicilin-type) and 11S (legumin-like) based on their sedimentation coefficients. The predominant lentil storage proteins, similar to other legume species, are salt-soluble globulins and water-soluble albumins (Table 3). Both the globulins and albumins of lentils are heterogeneous [29,30,31]. Legumes in general contain relatively large concentrations of globulins [32]. The 7S/11S ratio is an important characteristic for describing seed nutritional quality [33] and is reported to be very high in lentil, close to three [31]. The ratio of three in lentil is twelve-fold higher than in seeds of pea and *Medicago truncatula*. These results suggest that lentil may meet certain criteria for specific end uses based on protein quality characteristics.

Table 3 shows the reported high variation for each specific lentil seed storage fraction, particularly for albumins. The variation may reflect genotypic variation in storage protein profiles, or the extraction conditions employed in each individual study. For instance, Bhatty et al. [35] reported that, in addition to the albumin proteins, direct extraction of lentil meal with water solubilizes some non-protein nitrogen and may solubilize some salt-soluble proteins. Exhaustive extraction of the meal with a salt solution followed by precipitation of the salt-soluble proteins by dialysis provides a better estimate of this protein fraction.

Metabolic proteins (enzymes and structural proteins) are another major type of protein found in lentil seeds. Several non-storage proteins such as enzymes involved in DNA replication, proteins involved in various physiological processes and house-keeping proteins have been identified [31]. Sulieman et al. [37] showed that lentil protein fractions are altered quantitatively and qualitatively due to cooking, and the effect was most pronounced in prolamin fractions.

## 4. Amino Acids (AA)

Cultivated lentil proteins, like those of other grain legumes, are rich in endogenous amino acids (arginine, aspartic and glutamic acids, and leucine-more than half of total AA), low in some essential amino acids (EAA) like threonine, methionine, phenylalanine, tryptophan, histidine, valine, isoleucine, and leucine-excluding lysine), and poor in sulphur-containing amino acids (methionine and cysteine, see Table 4). For the WHO/FAO/United Nations University [40]. AA requirement patterns also showed low levels of both sulphur-containing amino acids and tryptophan in lentil seeds.

**Figure 1:**
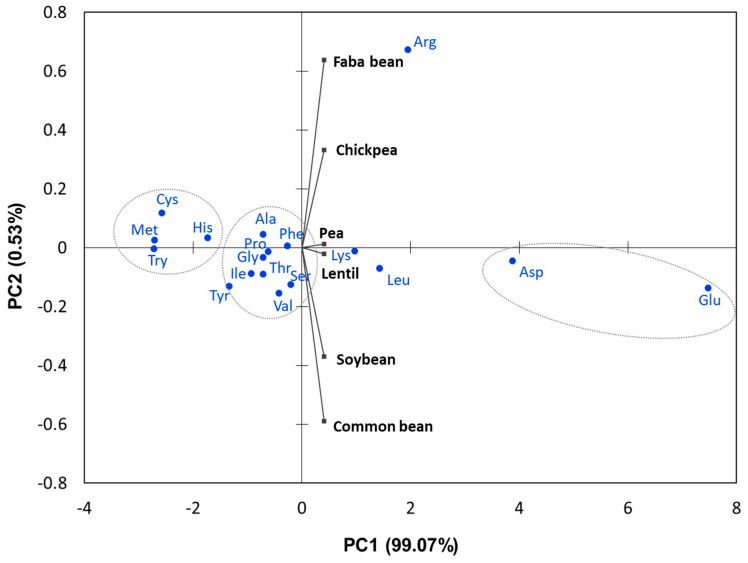
Principal component analysis (PCA) of amino acid data (meta-analysis) from Table 2. The biplot shows amino acid data and legume crops as vectors. Vectors that are close together are correlated in terms of the observed amino acid pool for each crop. PCA analysis was employed to illustrate relationships between amino acids and legume species using the R statistical package (R Development Core Team, 2018, www.Rproject.org).

Lentil has relatively a similar AA profile in comparison to other grain legume species (Table 4). Principal component analysis (PCA) of the AA from the studied legume species (Figure 1) revealed lentil and pea have very similar AA composition compared to other studied species and were characterized by high lysine. Principal component 1 alone explained over 99% of total variation. Faba bean was characterized by high arginine content. The PCA analysis also revealed that AA were grouped based on their AA amount. The average AA composition of *M. truncatula* was found to be very close to pea in various growing conditions [67]. Soybean and common bean had also similar AA profile (Figure 1).

Lentil protein is low in methionine (0.9%), especially when compared with animal-based proteins (>2.2%, [68]). Methionine is typically lower in plant-based proteins compared with animal-based proteins [69], and in general, plant proteins are only low in a few EAA. Combining plant proteins that are lower in lysine and higher in methionine (e.g., wheat, rice and hemp) with plant proteins that are higher in lysine and lower in methionine (grain legumes, including lentil) may balance the anabolic properties of plant-based protein intake [68].

The main non-protein AAs in lentil seeds are trigonelline [70], erythro-γ-hydroxyarginine 2(S), 4(R)-4-hydroxyarginine [71], γ-hydroxyarginine, γ-hydroxyornithine, and homoarginine [72].

## 5. Wild Lentil Taxa Protein Properties

The genus *Lens* has seven closely related taxa, namely *L. culinaris* (cultivated lentil), *L. orientalis*, *L. tomentosus* (primary gene pool); *L. odemensis*, *L. lamottei*, (secondary gene pool); *L. ervoides* (tertiary gene pool); and *L. nigricans* (quaternary gene pools) [73]. Bhatty [74] reported similar protein content for three wild lentil species from different gene pools (*L. orientalis*, *L. ervoides*, and *L. nigricans*) in comparison to cultivated lentil (range of 24.2%–26.2%). However, a much wider range (18.1%–32.7%) for protein content was reported recently for *L. orientalis*, *L. tomentosus*, *L. odemensis*, *L. ervoides*, and *L. nigricans*. The highest protein content was found in the *L. ervoides* accession, ILWL 47, with 32.7% protein content [15]. *L. ervoides* accessions also showed the highest variation compared to other studied species.

With respect to the storage protein fractions, all the wild species except *L. nigricans* had greater amounts of albumin and globulin than cultivated lentil. *L. nigricans* also had higher non-protein nitrogen than *L. orientalis* and *L. ervoides* [74]. In contrast, Rozan et al. [70] findings indicated that *L. ervoides* contained less non-protein AA than other *Lens* species.

The protein AA composition profiles of wild lentil species *L. orientalis*, *L. ervoides*, and *L. nigricans* were identical and similar to cultivated lentil [74]. A more recent study revealed that *L. orientalis* seeds had relatively higher AAsthan the other *Lens* species, including cultivated lentil [70].

## 6. Anti-Nutritional Factors Affecting Lentil Protein

Nutritional value of seed protein is determined primarily by the amount of anti-nutritional factors (ANFs) and the AA digestibility [75]. Removal of ANFs is necessary to improve nutritional quality. In general, whole lentils are low in ANFs. Lentil has relatively high tannin content compared to other grain legumes [7]. Tannins are primarily located in the seed coat and can be removed by processing (e.g., dehulling). Tannins can reduce protein digestibility by reacting with lysine and methionine and making them available in a smaller amount during digestion [76]. Zero tannin lentils are now available [77]. The zero tannin trait in lentil is controlled by a single recessive gene (*tan*) that results in a phenotype characterized by green stems, white flowers, and thin and transparent seed coats, a consequence of a major reduction in most of the seed coat polyphenols [78].

Trypsin inhibitors are low molecular weight proteins found in a wide range of plants including legumes [79] that irreversibly inhibit physiological trypsin enzyme. They induce hypersecretion of pancreatic enzymes (trypsin), thereby stimulating pancreatic hypertrophy, which leads to reduced digestion and absorption of amino acids and, hence, their bioavailability [79,80,81]. The bioavailability changes result in lower retention of nitrogen and sulphur, and impaired growth [82]. Trypsin inhibitor content of lentil is significantly lower than other grain legumes excluding pea (e.g., [83,84,85]). The trypsin inhibitors can be markedly reduced by soaking, cooking, and germination of lentil seeds [85].

## 7. Environmental Effects on Seed Proteins

The seed filling stage involves mobilization and transport processes required for importing various seed constituents. Environmental stresses (such as drought) can impair seed filling due to the disruption of metabolic pools downward of sucrose in starch synthesis [86], resulting in an increase of seed protein. Studies in many crops report increased content of seed protein in response to drought stress, for example, in wheat [87,88], chickpea [89], and soybean [90,91]. In contrast, some other studies reported reduction of protein content in response to other environmental stresses [92]. These differences may be attributable to the intensity and duration of stresses imposed on plants. Additionally, at the beginning of seed filling stage under unfavorable conditions, proteins related to protection against stress are probably synthesized (increasing protein content), whereas a reduction in protein content is due to their hydrolysis and degradation.

In lentil, heat stress is reported to reduce protein content (26%–41% [93]). Heat stress also inhibited the accumulation of globulins, albumins, glutelins, and prolamins. Excluding proline, glycine, alanine, isoleucine, leucine, and lysine, which increased under heat stress, the rest of the amino acids significantly decreased [93]. The decrease in lentil storage proteins and most of the AA composition profile resulting from high temperature stresses may be explained by the inactivity of biosynthetic enzymes [94], and by changes in nitrogen content [95]. The increase in some AA (e.g., proline and glycine) under stress conditions may be the effects of osmoregulation mechanisms [96]. Further investigation is required to determine the impacts of environmental stresses on protein content, protein fractions and AA composition, especially in legume crops such as lentil.

## 8. Yield and Protein Relationships and Stability

For lentil breeders, a major challenge is the simultaneous increase of both yield and protein content while maintaining progress in the development of resistance to biotic and abiotic stresses. Hamdi et al. [14], using two large sets of ICARDA lentil germplasm (829 + 987 accessions), found negative correlation between seed protein content and seed yield. They also reported high heritability (0.84) for protein content. Erskine et al. [16] showed the same trend in lentil for a smaller germplasm set. Later, Stoddard et al. [17] showed lack of correlation between protein and yield. More recently, Lizarazo et al. [97] reported negative relationships between protein concentration and seed yield in 14 lentil cultivars grown in a boreal growing environment. This may suggest that independent selection of both characters during breeding is challenging, i.e., the rate of gain in one trait being reduced by that in the other.

Barulina [12] indicated that the protein content varied little across locations among lentil accessions. Similar results are reported for lentil from different authors [17,35,97] and for other legume crops (e.g., [67,98]). These observations suggest there is low G × E interaction for protein content and AA composition. This supports the hypothesis that the nitrogen fixing ability of legumes makes their protein concentration relatively stable across environments [17]. It is known that the protein content of the seed is highly affected by soil nitrogen level, so in legumes the *Rhizobium* bacteria may greatly enhance the percentage seed protein. For example, Ivanov [99] reported large differences between seed protein content of non-inoculated and inoculated chickpea plants, 12.6% and 31.2%, respectively.

## 9. Agronomic Protein Yield of Lentil

Protein yield is calculated as protein fraction × grain yield. In lentil, a value of 0.33 t ha^−1^ protein was reported by Erskine et al. [16]. Khatun et al. [100] reported 0.2 t ha^−1^ for lentil protein yield grown in Bangladesh. Lizarazo et al. [97] reported protein yield of 0.4 t ha^−1^ for lentil grown in northern Europe, which was less than that for faba and (1.6 t ha^−1^) and narrow-leafed lupin (1.1 t ha^−1^). In pea, protein yield has been reported at 0.7 t ha^−1^ [101] and 0.9 t ha^−1^ [102].

The potential protein yield of lentil in specific environments has most likely not been fully explored due to limitations imposed by the narrow genetic base of most lentil breeding programs, which are not able to fully exploit the genetic potential due to adaptation bottlenecks. With the recent increasing emphasis on genomics within breeding programs, it may be possible to more fully explore the genetic potential for improvements in protein quantity and quality. The AGILE (Application of Genomic Innovation in the Lentil Economy) project (https://knowpulse.usask.ca/study/2675314), which evaluated phenotypic influences of temperature and photoperiod of 324 diverse lentil genotypes in replicated trials in the three main global agro-ecological regions of lentil production, may provide deeper understanding of aspects of protein quality and protein yield potential of lentil. Genomic information of the 324 sequenced lentil genotypes grown across the three major agro-ecological zones for lentil production may further provide the understanding of the underlying genetics of protein quality and protein yield in lentil.

## 10. Seed Crude Protein Determination in Lentil

The two most prevalent protein determination methods, Kjeldahl and Dumas combustion, are commonly used for grain and seed protein analysis in crops including lentil [12,17,19]. The methods rely on the release of nitrogen from the amine groups found in the peptide bonds of the polypeptide chains of protein. The traditional Kjeldahl method is based on oxidation to release nitrogen, while the Dumas combustion method breaks down the bonds in the peptide chains, permitting the release of nitrogen through complete combustion of the sample [103]. The released nitrogen content is multiplied by a factor to measure protein content [104]. The factor varies between crop species depending on nitrogen content of protein between 13% and 19%. For pulses, the average nitrogen (N) content of protein was found to be about 16%, which led to use the calculation N × 6.25 to convert nitrogen content into protein content [105].

In a series of experiments, most researchers employed a Kjeldahl method to determine crude protein in lentil. For example, Barulina [12] and Hawtin et al. [13] determined seed protein content in lentil accessions using a macro–Kjeldahl (Table 2). Recently, different versions of a modified Kjeldahl method were adopted to measure protein content in lentil seeds [15,17]. Although the Kjeldahl method was more precise and frequently used to analyze protein, this method is under threat by the challenge of safer, clearer, and faster instruments employed in the Dumas Combustion method [103] which resulted in faster, safer, and more reliable data for protein content in seeds compared to the Kjeldahl method. The Dumas combustion method was adopted to determine lentil seed protein content by Tahir et al. [8] when they compared lentil protein with other pulses in Canada using AACC (American Association of Cereal Chemists) method 46–30 to determine percent crude protein (CP; N × 6.25) through the use of a LECO CNS-2000 Nitrogen Analyzer (LECO Corporation, St. Joseph, MI, USA, Model No. 602-00-500). A rapid test method using near–infrared (NIR) spectroscopy as a complement to current protein determination using either the Kjeldahl or Dumas combustion method was also successfully applied to estimate protein content in lentil seeds using a different model of analyzer [14,16,19]. Protein measured through NIR was validated by calibrated value against measurements obtained through either Kjeldahl or Dumas methods using representative samples. The NIR method was found to be a rapid, low cost, and green complementary technique as it does not use chemicals and reagents [106]. NIR is a useful high throughput method for estimating protein content for lentil breeding programs if calibrated curves are used to validate the method. The value of protein concentration using three different methods in lentil research are illustrated in Table 2.

## 11. Protein Isolation Methods and Extraction

Lentils are traditionally consumed as whole seed, dehulled split seeds, or as footballs (cotyledons remain attached) in salads and soups or stews commonly known as ‘dal’ [4,107]. Diverse and novel applications are needed to identify ways to increase the use of lentils in the food industry. Nutritional components in lentil seeds such as dietary fiber, starch and protein concentrates or isolates can be extracted and separated [38]. These can be used as ingredients in the preparation of diverse value-added food products.

Isolation or separation of seed proteins from pulses is possible using wet or dry processes [108,109,110]. Dry processes, such as pin milling and then air classification, are designed to differentiate fractions of starch and protein based on size and density. Air classification separates milled lentils into a light to fine fraction (the protein concentrate) and a heavy or coarse fraction (the starch concentrate) [111,112]. Protein concentrates produced by air classification through dry processes generally contain 38%–68% protein [113,114]. In the past, air classification processes were well adapted to extraction of isolates of lentils and peas because of the large diameter and fairly uniform distribution of starch granules [112]. The dry method is a relatively easy and simple process, however, efficacy of separation is not high enough to yield high protein concentration. Currently, the wet method for extraction is more widely adopted for legume protein extraction [108]. The extraction of pulse proteins through wet methods may be relatively easy and reliable, as they are highly soluble under alkaline and acidic conditions. In wet methods, protein is extracted by solubilization in an alkaline solution by dispersing pulse flour in water at pH 8–10, followed by stirring of the dispersion. Then, the insoluble material is removed by centrifugation and proteins are recovered by adjusting the, supernatant pH to a value around 4.5, where proteins are precipitated isoelectrically. The precipitation is usually carried out at the isoelectric point of the protein at which its solubility is the lowest, which for lentil protein is around pH 4.5. The final concentrate or isolated protein is then dried using spray-drum or freeze-drying methods [115]. Lentil isolates prepared with an alkaline process yield overall 80% of protein [108,116,117]. Many researchers have used various wet fractionation methods to isolate lentil protein [108,109,116,117,118] under single or multiple isoelectric pH conditions using diluted sodium hydroxide [36] (Table 5). For example, Boye et al. [38] reported they extracted lentil protein isolates from red and yellow cotyledon lentils using isoelectric precipitation at pH 9 and 25 °C using a 1:10 solid to solvent ratio, resulting in protein concentrates between 78.2% and 88.6%. Similarly, Joshi et al. [119] extracted lentil protein isolate by alkaline extraction at pH 8 using 1:10 solid to solvent ratio at room temperature when they studied physicochemical characteristics of the isolated protein that obtained from three drying methods (freeze, spray and vacuum drying). In another study, Kaur et al. [120] revealed that yield of lentil protein isolate ranged from 81.7%–83.5% for Indian cultivars when they performed the protein isolation using isoelectric precipitation pH 4.5. In contrast, Alsohaimy et al. [117] found the highest protein recovery (93% and 100%) from lentil isolate at isoelectric pH of 12 with ammonium sulphate and alcohol precipitation solvent, with a 5:100 solid to solvent ratio. They compared seven different pH values ranging from 6 to 12 with three different protein recovery methods—isoelectric precipitation, ammonium sulphate preparation, and alcohol precipitation. Similarly, Lee et al. [115] reported pH 9 at 30 °C as the optimum extraction condition for green lentil that yielded 56.6% protein, and pH 8.5 at 35 °C for red lentil that yielded 59.3% protein when they compared five pH levels (distilled water, pH 8, 8.5, 9, and 9.5) and four temperatures (22, 30, 35, and 40 °C). Johnston et al. [121] used a modified isoelectric precipitation procedure by adjusting initial pH to 9 initially and then collecting lentil protein isolate at pH 4.6 with 1:10 solid to solvent ratio. Cultivar, particle size of the flour, type of solubilizing agent, temperature, and pH of extraction medium influenced the protein yield and quality [110].

In addition to air classification, a new emerging dry fractionation method, triboelectrostatic separation has emerged recently as a novel solvent-free approach to separate protein isolates in the food industry [123,124,125]. This method relies on differences in dielectric properties of flour particles instead of their size and density. The basic principle of this technique is that proteins can be electrostatically charged more than carbohydrates, because of the ionizable N-terminus and C-terminus groups in their amino acid residues [126]. Thus, an electric field can separate protein and carbohydrate rich fractions depending upon their types and magnitudes of charge. Like air classification, the main advantages of this method are that it does not use chemical reagents that render the concentrates unsafe for consumption, or that induce changes in functional character. This method is more energy efficient and effective than air classification because it effectively separates particles that are similar in size and density, but different in charge. The major limitation of this approach is that different components may exhibit similar charges under certain conditions, which reduces concentrate purity, and gravitational force may cause airborne particles [125].

Triboelectrostatic separation of legume flours pneumatically conveys the milled particles through tubes or fluidized/vibrating beds, thus imparting a positive or negative charge to the surface of the constituent protein and carbohydrate particles depending on their tribo-charging behavior and the contact medium. Upon contact charging, the oppositely charged particles are separated in a strong electric field [126]. The amount of charge gained on a particulate from triboelectric charging depends on factors such as surface conditions, area of contact, speed of rubbing, the materials involved, and humidity [123]. Electrostatic separation methods have been widely used in the mining and pharmaceutical industries, and the effectiveness of this approach is under investigation in the legume industry. For example, the development of optimization of triboelectric bio-separation using a single-stage separation of navy bean flour was successful [126]. Later, single- and multi-stage tribo-electrostatic bioseparation processes for dry fractionation of protein concentrate found that the two-stage approach resulted in a protein-rich fraction yield of 38% accounting for 60% of the total protein which was a significantly higher than that of the optimized single-stage triboelectrostatic separation [127]. Similarly, Jafari et al. [124] and Tabtabaei et al. [128] examined the physiochemical and functional properties of navy bean protein concentrated using triboelectrostatic separation. They found that electrostatically separated increased from 25.4% to 43.0% total protein yield of original navy bean flour, the protein fractions protein fractions exhibited superior solubility, superior emulsion stability, foam expansion and foam volume stability compared to the wet-fractionated navy bean protein isolate. This method has been extensively explored for fractionation of protein isolated from navy bean and other legumes, but there is limited information in the literature regarding the fractionation of protein from lentil flour using triboelectrostatic processes. However, the variation in turbocharging behavior of proteins and carbohydrates of flour extends its scope to protein isolation of lentil flours.

## 12. Bioactive Peptides

Food proteins not only provide dietary amino acids but also supply health benefits because of the presence of bioactive peptides, short fragments of 2–20 amino acid residues that are encrypted and inactive within the sequence of the precursor protein. Bioactive peptides play an important role in human health, being released during digestion or food processing (enzymatic hydrolysis, cooking, germination, fermentation, and ripening of foods), then absorbed in the intestine and transported to target tissues where they exert specific physiological effects [129].

Proteins from pulses are considered a good source of bioactive peptides. As an example, lentil convicilin was investigated as a source of bioactive peptides using the predictive tools in the BIOPEP database (http://www.uwm.edu.pl/biochemia, see Supplementary Table S1). This storage protein contains a total of 126 peptides encrypted in the amino acid sequence. The following array of biological effects was reported: (i) inhibition of angiotensin I converting enzyme (ACE), dipeptidyl aminopeptidase III and IV, calmodulin-dependent nucleotide phosphodiesterase (CaNPDE), and renin; (ii) stimulation of the release of vasoactive substances and the uptake of glucose; (iii) antioxidant activity, and (iv) regulation of secretion of gastric mucosa. This fact encourages more research studies exploring the biotechnological production of bioactive peptides from lentil proteins for functional food or nutraceutical applications.

A few studies are reported with the aim of ensuring efficient peptide release from lentil proteins. Critical processing parameters, such as pH, temperature and time require optimization, and the enzymes or microorganisms used for peptide release require evaluation for efficacy, reproducibility and stability. Enzymatic hydrolysis of lentil proteins has been performed using a wide number of proteolytic enzymes including Savinase, Alcalase, Protamex, Neutrase, Flavourzyme, bromelain, and papain [130,131]. Savinase^®^ 16L was reportedly the most effective enzyme to produce bioactive peptides from cultivated lentil concentrates [130].

Bioactive peptides derived from lentil proteins reportedly exhibit antihypertensive, antioxidant, and antifungal activities. Table 6 summarizes the in vitro effect of protein hydrolysates and bioactive peptides produced during gastrointestinal digestion, enzymatic hydrolysis, germination and fermentation of different lentil-based raw materials. Most studies performed to date have shown that enzymatic hydrolysis of lentil proteins by food grade commercial proteases (savinase, papain, alcalase, flavourzyme, and bromelain), digestive enzymes (pepsin, trypsin, α-chymotrypsin, pancreatin) or germination of lentil seeds (30–40 °C for 5 days) produce peptides with the ability to inhibit angiotensin I converting enzyme (ACE, EC. 3.4.15.1) (see Table 6). ACE is a carboxypeptidase involved in the cleavage of angiotensin I into angiotensin II, a vasoactive peptide that binds with receptors on the vascular wall to cause vasoconstriction, therefore, inhibition of ACE may reduce systolic and diastolic blood pressure [132].

Cultivated lentil proteins treated with Savinase^®^ produce multifunctional peptides with dual antioxidant and ACE inhibitory activities [133]. Three peptides were identified to have the highest potencies for inhibiting ACE and delay oxidation of proteins in the presence of oxygen radicals in vitro (LLSGTQNQPSFLSGF, NSLTLPILRYL, TLEPNSVFLPVLLH). The gastrointestinal digestion of these peptides greatly improved their dual biological activity, indicating that smaller peptide fragments with higher biological potency are produced at the gastrointestinal level. The antioxidant/antihypertensive activity of lentil peptides was linked to the primary structure of the C-terminal heptapeptide [133]. In particular, the ACE inhibition relies on the formation of hydrogen bonds between C-terminal residues of peptides and residues of the ACE catalytic site. The ability of these peptides to inhibit ACE is consistent with earlier studies showing that hydrophobic or aromatic residues or proline residue at the C-terminus positively contribute to the improvement of ACE inhibitory potency [134].

Lentil proteins are also sources of antifungal peptides with potential application as ingredients in the bakery industry. Recently, Rizzello et al. [135] produced a hydrolysate from a legume flour blend consisting of lentil, pea and faba bean by the combination of fermentation with *Lactobacillus plantarum* 1A7 and enzymatic hydrolysis with Veron. Among the antifungal compounds of the hydrolysate, four were identified as antifungal peptides derived from lentil lectin. These were purified to confirm their capacity to inhibit the development of *Penicillium roqueforti* conidia at a minimum inhibitory concentration of 7–9 mg/mL. Similarly, Wang and Ng [136] isolated a natural antifungal peptide from a red lentil protein extract by chromatographic fractionation. It was able to inhibit 50% of the mycelial growth of *Mycosphaerella arachidicola* at a concentration of 36 μM.

Although bioactive peptides have been identified and isolated from lentil for potential use in functional food and nutraceutical applications, none are currently available in the market for human use. The principal obstacle to the regulatory approval of health claims is the lack of in vivo studies supporting the health and safety claims of bioactive peptides [137]. To overcome these challenges, future research should focus more on generating data on the safety, efficacy, mechanisms of action, interactions of bioactive peptides with other drugs, absorption, distribution, metabolism, and excretion of bioactive peptides in clinical trials.

## 13. Food Applications of Lentil Proteins

Lentil protein ingredients represent a viable alternative to proteins from animal-derived sources and soybean, especially since the food industry aims to diversify their formulations because of cultural, religious or ethical dietary restrictions, growing populations, availability, and cost reduction. Although commonly sold as flours, lentil protein ingredients can also be further fractionated into higher protein enriched flours (<60% protein), concentrates (60%–85% protein) or isolates (>85% protein). Enriched flours and dry concentrates are usually produced by air classification methods [142], whereas concentrates/isolates are produced using wet extraction processes, such as by alkaline extraction followed by isoelectric precipitation [109]. These fractions are known to have good nutritional and functional value. In terms of functionality, the method and conditions used to produce the ingredients can have a big impact on their functionality within food applications.

### 13.1. Functionality

The majority of published studies have focused on the solubility and emulsifying properties of lentil proteins. Solubility of lentil proteins relates to the balance between protein-solvent and protein-protein interactions. Ladjal-Ettoumi et al. [143] reported a typical u-shaped pH-dependent solubility profile that was comparable to pea and chickpea proteins, where proteins assume a high surface charge away from their isoelectric point (net charge of 0 mV, pH 4.5) to promote more protein-solvent interactions (e.g., +30 mV at pH 2, and −40 mV at pH 8). Minimum solubility was found near the isoelectric point (~15%), and higher solubility at pH 2 and 8 (~65%). Can Karaca et al. [144] found lentil protein isolates at pH 7 to have high solubility (91%) when produced either from alkaline extraction-isoelectric precipitation or by salt extraction. Boye et al. [145] reported lentil protein concentrates produced by alkaline extraction-ultrafiltration showed greater solubility than those produced by alkaline extraction-isoelectric precipitation, with both showing the u-shaped pH-dependent profile.

Lentil protein isolates have demonstrated excellent emulsifying properties. During emulsion formation, proteins migrate to the oil-water interface and then rearrange to orient hydrophobic groups towards the oil phase and hydrophilic groups towards the water phase, lowering interfacial tension. Aggregation of absorbed proteins then creates a viscoelastic interfacial film to stabilize oil droplets from coalescence and gravitational separation. Ladjal-Ettoumi et al. [143] and Chang et al. [146] reported lentil protein-stabilized emulsions were most stable at pH levels away from their isoelectric point. Can Karaca et al. [144] indicated protein isolates prepared by alkaline extraction-isoelectric precipitation to have greater emulsion forming properties and stability than if salt extraction methods were used to prepare the isolates. Primozic et al. [147] examined the stabilizing effects of high-pressure homogenized lentil protein isolates relative to unmodified isolates. The authors found that homogenization acted to reduce the particle size, hydrophobicity, and interfacial storage moduli of the lentil proteins relative to unmodified proteins, but had no effect on their interfacial tension. Overall, modified lentil proteins showed better physical stability for prepared emulsions than unmodified proteins. Gumus et al. [148] showed that lentil protein-stabilized emulsions were also effective at inhibiting oxidative reactions within fish oil-in-water emulsions.

Water holding and fat/oil absorption capacities relates to the amount of water or oil that a gram of protein material can hold, and to the surface properties (hydrophilic vs. hydrophobic groups), protein/aggregate conformation and solvent conditions used. Aryee and Boye [149] reported both water holding and fat absorption capacities were improved with wet extraction (i.e., isolate), followed by cooked flour and then raw flour. Boye et al. [145] indicated that red lentil protein concentrates produced by alkaline extraction-ultrafiltration exhibited greater water holding and fat absorption capacities than concentrates produced by alkaline extraction-isoelectric precipitation. Water holding was similar to that of yellow pea, and greater than that of chickpea. Fat absorption capacity of red lentil protein concentrates (produced from alkaline extraction-ultrafiltration) was much greater than that of other pulses. In the case of foaming, proteins migrate to the air-water interface, re-align and aggregate similar as in emulsions, to form a viscoelastic lamella that entraps gas bubbles [39]. Toews and Wang [150] reported that lentil protein concentrate produced the most stable foams and had the highest foaming capacity in comparison to pea, navy bean and chickpea. Lately, investigation of the foaming properties of the pulse cooking water (known as aquafaba) is gaining some interest as an egg replacer [151]. This highlights an opportunity for food technologists to apply lentil proteins in food applications.

### 13.2. Challenges

Like other pulses, lentil protein ingredients also have unwanted flavour compounds that limit their widespread use. Numerous flavour reduction strategies are available to reduce these compounds in pulses. For instance, Chang et al. [152] used various organic solvent (acetone, ethanol, and isopropanol) treatments to reduce flavour compounds found in lentil protein isolates, however, this resulted in negative effects on protein functionality. Shariati-Levari et al. [153] used infrared heating to reduce flavour compounds in lentils, and Ma et al. [154] reported pre-cooking (roasting and cooking) significantly reduced flavour compounds in green lentils.

### 13.3. Applications

Lentil protein concentrates have been used to replace eggs in production of protein-enriched doughnuts [155], angel food cake, and muffins [156]. Lentil flour was used to make gluten-free crackers [157], lentil flour with transglutaminase has been used as a binding agent to make protein-enriched restructured beef steaks or beef patties [158,159], and lentil protein isolates have been used as an emulsifier to produce salad dressings [160]. Lentil protein isolates have also been applied as stabilizers for nano emulsion systems [147,161], as encapsulation agents for delivery of omega-3 rich oils [162,163], and in combination with zein, as anti-microbial films [164], and used to produce nanofibers [165]. Keeping in mind the many technological functions of lentil proteins, niches are emerging for their inclusion in functional foods, in nutraceuticals, or even in cosmetics.

## 14. Conclusions

Lentil is the most rapidly expanding pulse crop for direct human consumption, and has potential for greater impact as a desirable protein source for food applications. Improvements in lentil protein quality, amino acid composition, and processing fractions will enhance the nutritional quality of this rapidly expanding pulse crop. Genetic strategies focused on increasing the concentration of limiting amino acids are required in lentil. The potential of lentil wild species in breeding programs by introgression of favourable genes for protein improvement may have potential as a long-term breeding strategy.

## Figures and Tables

**Table 1:** Global trends of production of selected annual pulse crops in comparison to increases in soybean production and the global human population since 1960.

Crop	Global Production				Global Growth Trends	
	1960–1965		2012–2017			
	Annual Mean (Mt)	% of Total	Annual Mean (Mt)	% of Total	50 Years % Growth	Annual % Growth
Lentil	1.0	2.8	5.9	9.2	515	10.3
Faba bean	5.1	14.7	4.5	7.0	−12	−0.2
Pea	10.5	30.0	13.1	20.5	25	0.5
Chickpea	6.3	17.9	12.7	19.8	104	2.1
Common bean	12.1	34.5	27.9	43.5	132	2.6
Total pulses ^1,2^	34.9	100	64.2	90	84	1.7
Soybean (total)	28.6		319.0		1115	22.3
Soybean eaten	10.0		67.0		670	13.4
directly ^3^						
People (1967) ^4^	3465 million		7600 million		119	2.4

^1^ Total includes only the pulses listed in table. ^2^ Source: FAOSTAT (Food and Agriculture Organization of the United Nations) [9]. ^3^ Estimated direct human consumption. ^4^ Source: UN.

**Table 2:** Genetic variation and methods used measuring seed the crude protein content in cultivated lentil.

Protein Content (Range, % DM ^1^)	Number of Accessions	Environment	Method	Reference
27.5–31.7	-	Russia	A micro Kjeldahl Method	Barulina [12]
23.4–36.4	1688	Egypt	A micro Kjeldahl Method	Hawtin et al. [13]
25.5–28.9	24	Lebanon and Syria	NIR ^2^ using a Neotec model FQA51 A analyzer	Erskine et al. [16]
19.6–29.8 and 18.6–30.2	829 and 987	Tel Hadya (Syria)	NIR	Hamdi et al. [14]
23.9–26.3	58	Australia	A Kjeldahl N × 6.25 on an oven-dry basis method	Stoddard et al. [17]
23.0–32.0	-	-	-	Hedley [18]
24.3–30.2	4	-	NIR using a NIR Systems 6500 analyzer calibrated against the Dumas method	Wang and Daun [19]
23.8–29.3	22	Saskatoon, Canada	A Dumus Combustion method to determine Nitrogen percentage using a method in 46-30.01	Tahir et al. [8]
22.7–31.88	46	Turkey	A Kjeldahl method AOAC, Official Method of Analysis	Karaköy et al. [20]
21.8–27.1	14	Italy	A Kjeldahl method	Zaccardelli et al. [21]
25.3–29.3	35	Saudi Arabia	A Kjeldahl method of Association of Official Analytical Chemists (AOAC)	Alghamdi et al. [22]
24.6–30.0	23	Multiple	Multiple methods	Heuzé et al. [23]
10.5–27.1	45	India	A Kjeldahl method	Kumar et al. [15]

^1^ DM, dry matter. ^2^ NIR, near-infrared reflectance spectroscopy.

**Table 3:** Summary of reported analyses of cultivated lentil seed storage protein fractions (%) based on their solubility in different solvents.

Salt Soluble	Water Soluble	Acid Soluble	Ethanol Soluble	Reference
Globulins	Albumins	Glutelins	Prolamins	
44%	26%	20%	2%	Saint-Clair [34]
47%	4%	15%	3%	Bhatty et al. [35] ^1^
54%	20%	-	-	Bhatty [36]
42%	11%	47% ^2^	-	Neves and Lourenco [32]
27%	61%	3%	2%	Sulieman et al. [37]
70%	16%	11%	3%	Boye et al. [38]

^1^ About a quarter of the meal proteins were not solubilized by the solvents. ^2^ Prolamins, glutelins, non-nitrogen protein, and residual nitrogen. The quantitative data presented in this table are based on peptide bands and their molecular weight on SDS-PAGE (sodium dodecyl sulfate–polyacrylamide gel electrophoresis). The albumin, glutelin, and prolamin fractions contained 13, 4, and 10 polypeptides, with molecular weight (MW) of about 20, 17–46, and 16–64 kDa, respectively [39]. Globulins contain legumin- and vicilin-like proteins. The native globulin, with a molecular weight of 375 kDa, has twelve polypeptides and MW ranging from 14–61 kDa [32].

**Table 4:** Amino acid composition ± standard deviation (g/16 g N) in seeds of six major grain legumes.

Amino Acid	Lentil ^1^	Faba Bean ^2^	Pea ^3^	Soybean ^4^	Chickpea ^5^	Common Bean ^6^	SEM ^7^
Alanine	4.2 ± 0.4	4.1 ± 0.2	4.3 ± 0.2	4.2 ± 0.3	4.1 ± 0.5	3.8 ± 0.3	0.07
Arginine	7.8 ± 1.0	10.2 ± 1.1	8.2 ± 0.7	7.4 ± 0.6	9.0 ± 1.2	6.5 ± 0.7	0.51
Aspartic acid	10.7 ± 1.1	11.0 ± 1.6	11.3 ± 0.5	11.3 ± 0.7	11.6 ± 0.7	10.6 ± 1.3	0.15
Cysteine	1.1 ± 0.3	1.4 ± 0.3	1.3 ± 0.3	1.3 ± 0.4	1.3 ± 0.1	1.1 ± 0.2	0.06
Glutamic acid	16.1 ± 2.6	16.7 ± 2.2	16.4 ± 0.7	17.8 ± 1.2	16.8 ± 2.2	15.6 ± 2.1	0.31
Glycine	4.1 ± 0.7	4.3 ± 0.2	4.3 ± 0.2	4.1 ± 0.4	3.6 ± 0.6	4.2 ± 0.5	0.10
Histidine	2.4 ± 0.5	2.6 ± 0.2	2.3 ± 0.3	2.6 ± 0.1	2.6 ± 0.5	2.7 ± 0.2	0.05
Isoleucine	4.1 ± 0.5	4.0 ± 0.4	4.1 ± 0.5	4.4 ± 0.6	3.8 ± 0.4	4.2 ± 0.3	0.09
Leucine	7.2 ± 0.4	7.7 ± 0.6	7.3 ± 0.8	7.5 ± 0.4	7.0 ± 0.4	7.5 ± 0.7	0.11
Lysine	6.7 ± 0.6	6.4 ± 0.1	7.6 ± 1.2	6.4 ± 0.6	6.5 ± 0.8	6.3 ± 0.5	0.19
Methionine	0.9 ± 0.2	0.7 ± 0.1	1.0 ± 0.1	1.3 ± 0.3	1.4 ± 0.3	1.0 ± 0.4	0.11
Phenylalanine	5.0 ± 0.6	4.2 ± 0.2	4.8 ± 0.5	4.8 ± 0.3	5.5 ± 0.5	4.4 ± 0.7	0.18
Proline	3.8 ± 0.4	4.1 ± 0.5	4.4 ± 0.9	5.1 ± 0.3	4.4 ± 0.4	3.8 ± 0.4	0.19
Serine	4.7 ± 0.7	4.6 ± 0.4	4.9 ± 0.5	5.1 ± 0.5	4.8 ± 0.9	5.2 ± 0.7	0.08
Threonine	3.7 ± 0.4	3.5 ± 0.2	3.8 ± 0.3	3.9 ± 0.4	3.7 ± 0.6	4.0 ± 0.2	0.07
Tryptophan	0.8 ± 0.1	0.9 ± 0.1	1.2 ± 0.6	1.4 ± 0.3	1.0 ± 0.1	1.1 ± 0.4	0.09
Tyrosine	2.5 ± 0.7	3.1 ± 0.3	3.3 ± 0.5	3.4 ± 0.6	2.9 ± 0.5	3.7 ± 0.6	0.18
Valine	4.7 ± 0.4	4.4 ± 0.4	4.5 ± 0.5	4.7 ± 0.5	4.0 ± 0.4	4.9 ± 0.5	0.13

^1^ Mean of data extracted from Kahn and Baker [41]; Chatterjee and Abrol [42]; Bhatty et al. [35]; Sosulski [43]; Bhatty and Christison [44]; Shekib et al. [45]; Pirman et al. [46]; Porres et al. [47]; Zia-Ul-Haq et al. [48], and Grela et al. [7]. ^2^ Mean of data extracted from Kaldy and Kasting [49]; Bhatty and Christison [44]; Lisiewska et al. [50]; Schumacher et al. [51], and Grela et al. [7]. ^3^ Mean of data extracted from Bhatty and Christison [44]; Leterme et al. [52]; Pownall et al. [53]; Schumacher et al. [51], and Grela et al. [7]. ^4^ Mean of data extracted from Kuiken et al. [54]; Tkachuk and Irvine [55]; Cho and Bayley [56]; Cavins et al. [57]; Wang and Cavins [58]; Zarkadas et al. [59], and Sotak-Peper et al. [60]. ^5^ Mean of data extracted from Wang and Daun [61]; Alajaji and El-Adawy [62]; Wang et al. [63]; El-Beltagi et al. [64], and Grela et al. [7]. ^6^ Mean of data extracted from Wu et al. [65]; Słupski [66] and, Grela et al. [7]. The mean of AA for all species includes data from https://www.feedtables.com. ^7^ Standard error of means.

**Table 5:** Conditions for wet fractionation methods for extraction of lentil protein in six recent studies.

Conditions for Lentil Protein Extraction	% Protein Yield	% Protein in Final Extracts	Reference
pH: 6, 7, 8, 9, 10, 11, and 12 Temperature: room Solid to solvent ratio: 5:100	80.0	21.5	Alsohaimy et al. [117]
pH: 8, 8.5, 9, 9.5 Temperature: 22 °C, 30 °C, 35 °C, and 40 °C Solid to solvent ratio: 1:10	56.6–59.3	-	Lee et al. [115]
pH: 9 Temperature: 25 °C Solid to solvent ratio: 1:10	50.3–69.1	-	Boye et al. [38]
pH: 8 Temperature: room Solid to solvent ratio: 1:10	-	-	Joshi et al. [119]
pH: 4.6 Temperature: room Solid to solvent ratio: 1:10	82.0	14.5	Johnston et al. [121]
pH: 8, 9 and 10 Temperature: room Solid to solvent ratio: 1:10	70.3–85.7	12.3–16.5	Jarpa-Parra et al. [122]

**Table 6:** In vitro biological activity of cultivated lentil protein hydrolysates and peptides.

Biological Activity	Raw Material	Processing Conditions	Peptide Sequence	Effect Observed	Reference
Antioxidant and antihypertensive	Protein concentrate	Enzymatic hydrolysis with Savinase 16 L (0.1 U/mg protein, pH 8, 40 °C, 2 h)	LLSGTQNQPSFLSGF ^1^	ACE ^2^ inhibition: IC_50_^3^ = 120 µM ORAC ^4^: 0.013 µmol Trolox eq./µmol	Garcia-Mora et al. [133]
			NSLTLPILRYL	ACE inhibition: IC_50_ = 77.14 µM ORAC: 1.432 µmol Trolox eq./µmol	
			TLEPNSVFLPVLLH	ACE inhibition: IC_50_ = 117.81 µM ORAC: 0.139 µmol Trolox eq./µmol	
Antihypertensive	Sprouts	Germination (30–40 °C for 5 days, 98% humidity)	Unknown	ACE inhibition: IC_50_ = 0.044 and 0.034 mg/mL	Mamilla and Mishra [138]
Antifungal	Flour	Fermentation with *Lactobacillus plantarum* (7 log cfu/g) and enzymatic hydrolysis with Veron PS (E/S ^5^ of 1/400, 30 °C, 24 h)	HIGIDVNSIK	Inhibition of germination of *Penicillium roqueforti* DPPMAF1 conidia: MIC ^6^ = 7–9 mg/mL	Rizzello et al. [135]
			NLIFQGDGYTTK		
			FSPDQQNLIFQGDGYTTK		
			HIGIDVNSIK		
Antihypertensive	Protein isolate	Enzymatic hydrolysis with pepsin (E/S of 1/100, pH 2, 37 °C for 18 h)	Unknown	ACE inhibition: IC_50_ = 606 µg/mL	Boschin et al. [139]
Antihypertensive	Protein isolate	Pepsin (250 U/mg, pH 2, 37 °C, 2 h) and pancreatin (0.7%, pH 7, 37 °C, 1 h)	KLRT	ACE inhibition: IC_50_ = 0.13–0.02 mg/mL for different peptide fractions	Jakubczyk and Baraniak [140]
			TLHGMV		
			VNRLM		
Antihypertensive	Red protein concentrates	Pepsin (E/S of 1/250, for 2 h, pH 2, 37 °C) + Trypsin and α-chymotrypsin (E/S of 1/250 for each enzyme, 2.5 h, pH 6.5, 37 °C)	Unknown	ACE inhibition: IC_50_ = 0.090 mg/mL	Barbana and Boye [131]
		Papain (E/S of 1/25, pH 6.5, 4 h, 40 °C)	Unknown	IC_50_ = 0.086 mg/mL	
		Alcalase (1/8 for E/S ratio, pH 7, 1 h, 50 °C) + Flavourzyme (E/S of 1/10, pH 8, 1.5 h at 50 °C)	Unknown	IC_50_ = 0.154 mg/mL	
		Bromelain (E/S of 1/4, pH 8, 8 h, 40 °C)	Unknown	IC_50_ = 0.190 mg/mL	
	Green protein concentrates	Pepsin (pepsin (E/S of 1/250, for 2 h, pH 2, 37 °C) + Trypsin and α-chymotrypsin (E/S of 1/250 for each enzyme, 2.5 h, pH 6.5, 37 °C)	Unknown	ACE inhibition: IC_50_ = 0.053 mg/mL	
		Papain (E/S of 1/25, pH 6.5, 4 h, 40 °C)	Unknown	IC_50_ = 0.080 mg/mL	
		Alcalase (1/8 for E/S ratio, pH 7, 1 h, 50 °C) + Flavourzyme (E/S of 1/10, pH 8, 1.5 h at 50 °C)	Unknown	IC_50_ = 0.152 mg/mL	
		Bromelain (E/S of 1/4, pH 8, 8 h, 40 °C)	Unknown	IC_50_ = 0.174 mg/mL	
Antihypertensive	Red protein isolates	Pepsin (E/S of 4/10, pH 2, 37 °C, 2 h) and pancreatin (E/S of 0.5/10, pH 7, 37 °C, 2 h)	Unknown	ACE inhibition: IC_50_ = 0.008–0.33 mg/mL in gastric phase	Akıllıoğlu and Karakaya [141]
				IC_50_ = 0.26–0.89 mg/mL in intestinal phase	
Antifungal	Red lentil extract	Chromatographic fractionation	TETNSFSITKFSPDGNKLIFQGDGYTTKGK	Inhibition of mycelial growth in *Mycosphaerella arachidicola*: IC_50_ = 36 μM	Wang and Ng [136]

^1^ Amino acids are coded according to their one letter abbreviation: A = alanine; C = cystine, D = aspartic acid, E = glutamic acid, F = phenylalanine, G = glycine, H = histidine, I = isoleucine, K = lysine, L = leucine, M = methionine, N = asparagine, P = proline, Q = glutamine, R = arginine, S = serine, T = threonine, V = valine, W = tryptophan, Y = tyrosine. ^2^ ACE, angiotensin I converting enzyme. ^3^ IC_50_, inhibitory concentration that reduces 50% of the original enzymatic activity. ^4^ ORAC, oxygen radical absorbance capacity. ^5^ E/S, enzyme to substrate ratio. ^6^ MIC, minimal inhibitory concentration.

## Supplementary Materials

The following are available online at https://www.mdpi.com/2304-8158/8/9/391/s1, Table S1: List of peptides with bioactive potential encrypted in lentil (*Lens culinaris* Medik.) convicilin (*see* http://www.uwm.edu.pl/biochemia).
