# Supplementary material for: Seed Protein of Lentils: Current Status, Progress, and Food Applications
Source: Foods. 2019 Sep 4;8(9):391. doi: 10.3390/foods8090391 (PMC6769807; doi:10.3390/foods8090391)
Supplement: Supplementary file 1 [file foods-08-00391-s001.pdf]

Supplementary Table S1. List of peptides with bioactive potential encrypted in lentil (*Lens culinaris* Medik.) convicilin (*see* http://www.uwm.edu.pl/biochemia)

ID	Activity[1]	Number	Sequence[2]	Location
3460	antiamnestic	2	PG	[24-25], [45-46]
3380	ACE inhibitor	1	RY	[126-127]
3386	ACE inhibitor	1	KW	[72-73]
3537	ACE inhibitor	1	PR	[21-22]
3546	ACE inhibitor	1	VAY	[10-12]
3551	ACE inhibitor	2	LF	[148-149], [157-158]
3563	ACE inhibitor	1	AY	[11-12]
7582	ACE inhibitor	1	RP	[49-50]
7583	ACE inhibitor	1	AF	[6-7]
7585	ACE inhibitor	1	LA	[5-6]
7586	ACE inhibitor	1	KR	[113-114]
7587	ACE inhibitor	1	VP	[23-24]
7589	ACE inhibitor	1	YA	[12-13]
7593	ACE inhibitor	1	IF	[3-4]
7596	ACE inhibitor	1	GI	[1-2]

7603	ACE inhibitor	2	GR	[30-31], [69-70]
7607	ACE inhibitor	2	GS	[18-19], [92-93]
7610	ACE inhibitor	2	GQ	[25-26], [46-47]
7611	ACE inhibitor	2	GK	[53-54], [131-132]
7614	ACE inhibitor	2	HG	[52-53], [96-97]
7615	ACE inhibitor	2	GE	[34-35], [88-89]
7618	ACE inhibitor	1	SG	[68-69]
7620	ACE inhibitor	1	GD	[97-98]
7622	ACE inhibitor	4	EG	[17-18], [33-34], [87-88], [130-131]
7623	ACE inhibitor	1	EA	[65-66]
7625	ACE inhibitor	2	PG	[24-25], [45-46]
7680	ACE inhibitor	1	QK	[63-64]
7692	ACE inhibitor	1	KF	[153-154]
7698	ACE inhibitor	1	NK	[152-153]
7741	ACE inhibitor	2	RR	[125-126], [143-144]
7743	ACE inhibitor	1	KA	[36-37]
7829	ACE inhibitor	2	VE	[83-84], [116-117]
7830	ACE inhibitor	1	TE	[120-121]

7838	ACE inhibitor	1	EW	[89-90]
7840	ACE inhibitor	6	EK	[35-36], [60-61], [71-72], [80-81], [110-111], [122-123]
7841	ACE inhibitor	5	KE	[54-55], [61-62], [64-65], [76-77], [132-133]
8382	ACE inhibitor	1	RYQ	[126-128]
9039	ACE inhibitor	1	IFL	[3-5]
9173	ACE inhibitor	2	RG	[29-30], [91-92]
3285	antithrombotic	2	PG	[24-25], [45-46]
3354	antithrombotic	2	DEE	[57-59], [78-80]
3351	stimulating vasoactive substance release	4	EEE	[58-60], [84-86], [85-87], [133-135]
8325	Glucose uptake stimulating	1	II	[2-3]
8329	stimulating vasoactive substance release	13	EE	[55-56], [58-59], [59-60], [79-80], [84-85], [85-86], [86-87], [100-101], [109-110], [117-118], [121-122], [133-134], [134-135]
8330	stimulating vasoactive substance release	2	SE	[19-20], [138-139]
2890	neuropeptide	2	GQ	[25-26], [46-47]
2754	regulating the stomach mucosal membrane activity	2	PG	[24-25], [45-46]
7866	antioxidative	1	AY	[11-12]
8058	antioxidative	1	RHG	[95-97]

8065	antioxidative	1	RHR	[106-108]
8134	antioxidative	1	KD	[123-124]
8474	antioxidative	1	RYQ	[126-128]
9363	antioxidative	1	NEN	[160-162]
8249	CaNPDE[3] inhibitor	1	KF	[153-154]
8248	Renin inhibitor	1	KF	[153-154]
9433	Renin inhibitor	1	YA	[12-13]
4006	activating ubiquitin-mediated proteolysis	1	LA	[5-6]
3172	dipeptidyl peptidase IV inhibitor	1	VA	[10-11]
3174	dipeptidyl peptidase IV inhibitor	1	KA	[36-37]
3175	dipeptidyl peptidase IV inhibitor	1	LA	[5-6]
3181	dipeptidyl peptidase IV inhibitor	1	VP	[23-24]
3183	dipeptidyl peptidase IV inhibitor	1	VV	[82-83]
8505	dipeptidyl peptidase IV inhibitor	1	SP	[44-45]
8518	dipeptidyl peptidase IV inhibitor	1	RP	[49-50]
8529	dipeptidyl peptidase IV inhibitor	1	EP	[20-21]
8530	dipeptidyl peptidase IV inhibitor	1	NP	[145-146]
8555	dipeptidyl peptidase IV inhibitor	3	FL	[4-5], [147-148], [154-155]

8558	dipeptidyl peptidase IV inhibitor	6	EK	[35-36], [60-61], [71-72], [80-81], [110-111], [122-123]
8600	dipeptidyl peptidase IV inhibitor	1	WRG	[90-92]
8611	dipeptidyl peptidase IV inhibitor	1	WRP	[48-50]
8675	dipeptidyl peptidase IV inhibitor	2	WR	[48-49], [90-91]
8691	dipeptidyl peptidase IV inhibitor	1	WE	[73-74]
8758	dipeptidyl peptidase IV inhibitor	1	AE	[37-38]
8759	dipeptidyl peptidase IV inhibitor	1	AF	[6-7]
8765	dipeptidyl peptidase IV inhibitor	1	AY	[11-12]
8767	dipeptidyl peptidase IV inhibitor	1	DP	[98-99]
8769	dipeptidyl peptidase IV inhibitor	1	DR	[124-125]
8770	dipeptidyl peptidase IV inhibitor	4	EG	[17-18], [33-34], [87-88], [130-131]
8773	dipeptidyl peptidase IV inhibitor	1	ES	[139-140]
8774	dipeptidyl peptidase IV inhibitor	1	ET	[135-136]
8776	dipeptidyl peptidase IV inhibitor	1	EW	[89-90]
8781	dipeptidyl peptidase IV inhibitor	2	GE	[34-35], [88-89]
8785	dipeptidyl peptidase IV inhibitor	1	GI	[1-2]
8790	dipeptidyl peptidase IV inhibitor	1	HE	[129-130]
8794	dipeptidyl peptidase IV inhibitor	1	HR	[107-108]

8801	dipeptidyl peptidase IV inhibitor	1	II	[2-3]
8808	dipeptidyl peptidase IV inhibitor	5	KE	[54-55], [61-62], [64-65], [76-77], [132-133]
8809	dipeptidyl peptidase IV inhibitor	1	KF	[153-154]
8814	dipeptidyl peptidase IV inhibitor	1	KR	[113-114]
8815	dipeptidyl peptidase IV inhibitor	1	KS	[150-151]
8816	dipeptidyl peptidase IV inhibitor	1	KT	[111-112]
8817	dipeptidyl peptidase IV inhibitor	1	KV	[81-82]
8818	dipeptidyl peptidase IV inhibitor	1	KW	[72-73]
8824	dipeptidyl peptidase IV inhibitor	1	LT	[155-156]
8840	dipeptidyl peptidase IV inhibitor	1	ND	[14-15]
8841	dipeptidyl peptidase IV inhibitor	1	NE	[160-161]
8854	dipeptidyl peptidase IV inhibitor	1	PF	[146-147]
8855	dipeptidyl peptidase IV inhibitor	2	PG	[24-25], [45-46]
8862	dipeptidyl peptidase IV inhibitor	1	PS	[50-51]
8869	dipeptidyl peptidase IV inhibitor	2	QE	[32-33], [141-142]
8872	dipeptidyl peptidase IV inhibitor	1	QH	[128-129]
8877	dipeptidyl peptidase IV inhibitor	3	QS	[39-40], [43-44], [67-68]
8878	dipeptidyl peptidase IV inhibitor	1	QT	[119-120]

8879	dipeptidyl peptidase IV inhibitor	1	QV	[115-116]
8880	dipeptidyl peptidase IV inhibitor	1	QW	[47-48]
8882	dipeptidyl peptidase IV inhibitor	2	RG	[29-30], [91-92]
8883	dipeptidyl peptidase IV inhibitor	2	RH	[95-96], [106-107]
8885	dipeptidyl peptidase IV inhibitor	1	RK	[75-76]
8888	dipeptidyl peptidase IV inhibitor	1	RN	[144-145]
8889	dipeptidyl peptidase IV inhibitor	2	RR	[125-126], [143-144]
8892	dipeptidyl peptidase IV inhibitor	1	SH	[51-52]
8899	dipeptidyl peptidase IV inhibitor	1	TE	[120-121]
8904	dipeptidyl peptidase IV inhibitor	1	TK	[112-113]
8905	dipeptidyl peptidase IV inhibitor	1	TL	[156-157]
8910	dipeptidyl peptidase IV inhibitor	1	TS	[136-137]
8916	dipeptidyl peptidase IV inhibitor	2	VE	[83-84], [116-117]
8932	dipeptidyl peptidase IV inhibitor	1	YA	[12-13]
8943	dipeptidyl peptidase IV inhibitor	1	YQ	[127-128]
9485	dipeptidyl peptidase III inhibitor	2	RR	[125-126], [143-144]
9487	dipeptidyl peptidase III inhibitor	2	GE	[34-35], [88-89]
9489	dipeptidyl peptidase III inhibitor	1	PR	[21-22]

9491	dipeptidyl peptidase III inhibitor	1	RV	[22-23]
9499	dipeptidyl peptidase III inhibitor	1	LA	[5-6]
9502	dipeptidyl peptidase III inhibitor	3	FL	[4-5], [147-148], [154-155]
9504	dipeptidyl peptidase III inhibitor	1	PE	[99-100]
9505	dipeptidyl peptidase III inhibitor	1	PF	[146-147]
9511	dipeptidyl peptidase III inhibitor	1	KA	[36-37]

[1] Potential bioactivities of peptide sequences found in convicilin were retrieved from the bioactive peptide library of the BIOPEP database (http://www.uwm.edu.pl/biochemia/index.php/en/biopep)

[2] Amino acids are coded according to their one letter abbreviation: A = alanine; C = cystine, D = aspartic acid, E = glutamic acid, F = phenylalanine, G = glycine, H = histidine, I = isoleucine, K = lysine, L = leucine, M = methionine, N = asparagine, P = proline, Q = glutamine, R = arginine, S = serine, T = threonine, V = valine, W = tryptophan, Y = tyrosine

[3] CaNPDE = calmodulin-dependent cyclic nucleotide phosphodiesterase
